# A theoretical review on the interplay of EFL/ESL teachers' career adaptability, self-esteem, and social support

**DOI:** 10.3389/fpsyg.2022.915610

**Published:** 2022-08-12

**Authors:** Yuxiu Xue

**Affiliations:** School of Foreign Languages, Yancheng Institute of Technology, Yancheng, China

**Keywords:** psychology, self-esteem, social support, EFL/ESL teacher, career adaptability

## Abstract

Second/foreign language education has been considered a complex profession due to the interaction of numerous internal and external factors. Owing to such complications, the teaching profession is seen as a tough task, for which L2 teachers must be psychologically ready. To provide effective education, teachers need to have career adaptability to manage the challenges and the transition of roles in academia. This ability may be affected by many factors like teachers' self-esteem and perceived social support. Despite the significance of these three constructs, few (if any) studies have focused on their interaction. Against this lacuna, this study presented a theoretical review of the concepts, definitions, dimensions, and related studies to EFL/ESL teachers' CA, self-esteem, and social support. The study also offers some implications for teachers, trainers, school principals, and researchers trying to increase their awareness of psycho-social factors involved in L2 education.

## Introduction

It is widely believed by educators that second/foreign language education is an intricate process given the interactions among various cognitive and linguistic factors (Larsen-Freeman and Cameron, 2008). Moreover, the abundance of psycho-emotional, socio-cultural, and interpersonal traits multiplies the complexity of the teaching profession (Mercer, 2020; Gu and Sun, 2021). Additionally, the nested nature of these factors and the emotional pressures that L2 education places on teachers require psychological power and readiness among teachers (King and Ng, 2018; MacIntyre et al., 2019). To constantly improve in teaching, EFL/ESL teachers need to be adaptable to the transitions and changes occurring during their instruction. In other words, they must have career adaptability (CA), which concerns one's competencies in effectively managing work-related transitions and making proper adjustments at the workplace (Savickas, 2013). It also refers to a psychological capability to deal with both expected and unexpected events and challenges in teaching (Rottinghaus et al., 2005). CA is one of the most critical abilities for EFL/ESL teachers who are entering a profession full of complexities and setbacks. It helps them recover from difficulties, remain comfortable in the profession, and take their roles by adapting to changes and transitions (Chen et al., 2020). The construct has recently gained more scholarly attention in educational contexts given its impact on various aspects of teaching and learning (McIlveen et al., 2019; Green, 2021). Several studies examined numerous psycho-emotional and work-related variables such as satisfaction, motivation, emotional intelligence, optimism, hope, loyalty, and many more. They also suggest that the ability to deal with changes and challenges in teaching leads to quality improvement (Eryilmaz and Kara, 2017, 2018; McLennan et al., 2017).

Another domain that EFL teachers' CA can influence and have an interaction with is their self-esteem, which is an individual's attitude toward himself/herself (Burns, 1979). It is a perception of self-worth that has a significant role in almost all areas of education (Branden, 2001; Brown, 2007). The strong linkage that a perceived sense of worth has with other internal factors extended the scope of self-esteem research that substantiates its correlation with several L2 variables including, academic engagement, proficiency, language skills, achievement, motivation, intelligence, and the like (Kalanzadeh et al., 2013; Satriani, 2014; Lee et al., 2017). However, the influence and interplay of teachers' work-related and psycho-social constructs like CA on the construction and growth of self-esteem have been mostly overlooked in EFL/ESL contexts. It can be contended that teachers' sense of self-esteem can simultaneously be the cause, facilitator, and result of CA in that an EFL/ESL teacher's perceived self-value can lead to a higher level of adaptability to the teaching profession and CA, which, in turn, can facilitate or improve teachers' self-esteem as well. When a teacher is skillful in coping and adapting to changes and challenges of L2 education, his/her perceived self-esteem escalates, too.

Another psycho-social variable interacting with both CA and self-esteem is social support, which refers to the physical, affective, instrumental, and informational help that a person receives from his/her social relations (Lu et al., 2015). Social support includes many social interactions that one has with family members, friends, colleagues, and others (Siklos and Kerns, 2006). Various bodies of research show that social support influences teachers' psychological states, efficacy, and teaching quality (Shen, 2009; Skaalvik and Skaalvik, 2009; Minghui et al., 2018). It has also been identified to affect teachers' level of work engagement, burnout, and commitment (Minghui et al., 2018; Fiorilli et al., 2019). The social support that is received from a teacher's internal or external source can enhance his/her sense of self-worth and competence to adapt to professional transitions as well. Nevertheless, conducting theoretical and empirical studies on the interplay of EFL/ESL teachers' CA, self-esteem, and social support has not been sufficiently highlighted, to date. Against this shortcoming, the present mini-review article aimed to provide a theoretical analysis of the interaction among CA, self-esteem, and social support in the EFL/ESL context. It also presents the definitions, conceptualizations, dimensions, and related studies to each variable.

## Background

### The concept of career adaptability

Career adaptability has been considered a psychological ability for people to strike a balance among several factors and elements when they face a transition in their career (Chen et al., 2020). It is a very significant psycho-social construct related to one's transition from school life to career life (Koen et al., 2012; Savickas, 2013). According to Rottinghaus et al. (2005), CA is a representation of positive psychological capital (PPC) and resilience in teachers. In other words, CA is the ability to successfully adapt to professional challenges, career transitions, role changes, and recovery from traumatic experiences related to work and the workplace (Savickas, 2005, 2013; Chen et al., 2020). It can be regarded as a psychological resource or a dimension of resilience that protects teachers in occupational adversities (Luthans et al., 2007; Luthans and Youssef-Morgan, 2017). Through CA, using self-regulative psychological strengths, teachers can maintain and develop in their job the ability to deal with expectable and unexpectable problems and conditions in work and work roles (Savickas, 2005; Savickas and Porfeli, 2012).

### The dimensions of career adaptability

**Figure 1:**
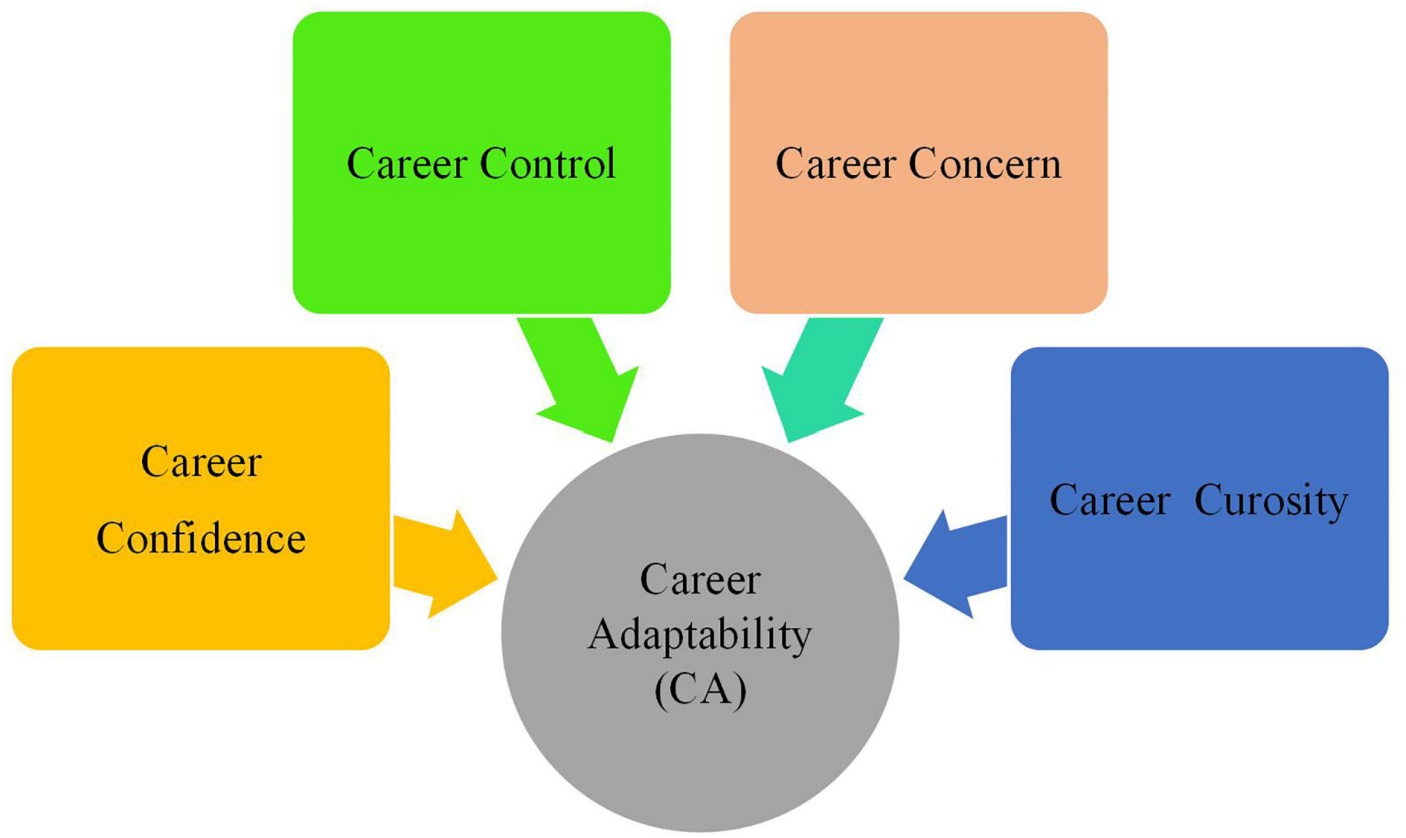
The dimensions of career adaptability (Savickas, 2005, p. 51).

CA is considered as a complex variable because various factors may influence it and its multiple dimensions. The growing body of research has pointed to four dimensions of CA (Figure 1), namely, (1) *career concern* (i.e., a person's planning skills for career development), (2) *career control* (i.e., one's capacity to make decisions and take charge of his/her career development), (3) *career curiosity* (i.e., one's ability to understand and learn about him/herself and the contextual aspects of career development), and (4) *career confidence* (one's problem-solving skills during his/her career development process (Savickas, 2005).

Based on these dimensions, Savickas (2013) maintained that individuals or professionals with higher levels of CA demonstrate more concern about future tasks associated with their job, take control of their own career growth, have the curiosity to unpack and identify possible selves and career prospects, and show confidence in coping with challenges of gaining success in their career-related tasks. All these dimensions affect and are affected by one's self-esteem or sense of self-worth in doing a task, which is described in the following section.

### The notion of self-esteem

Self-esteem or one's sense of self-worth is one of the most important psychological constructs in education. It refers to the degree to which a person perceives and considers worth or value for him/herself (Morin and Racy, 2021). Simply, it is a subjective evaluation (positive or negative) of one's worth (Smith and Mackie, 2007). Self-esteem is a flexible variable in educational psychology, on which numerous personal, contextual, cultural, affective, and social factors can influence (Kahng and Mowbray, 2004). The concept has been divided into various typologies. Based on the level, self-esteem has been classified into *inflated, high*, and *low* (Baumeister and Boden, 1998; Piff, 2014). People with inflated self-esteem always perceive themselves as worthy and outdo others or undervalue others' capabilities. On the contrary, people with high self-esteem are predisposed to appreciate and confirm themselves by believing in their own skills. However, individuals with low self-esteem are doubtful of themselves and their capabilities to accomplish a task. Therefore, they perform poorly and are stressed and anxious regularly. Going even further, Rosenberg et al. (1995) proposed another categorization for self-esteem. They divided the construct into *global* self-esteem and *specific* self-esteem based on the scope and coverage. Global self-esteem is a broad sense of self-worth in different realms, while specific self-esteem is observed in only a particular area of life or job.

### The cognate terms of self-esteem

Different cognates have been used in place of or synonymous with self-esteem. They encompass self-worth, self-concept, self-confidence, self-compassion, self-image, self-efficacy, and self-competence. Even if they seem analogous, they have different concerns. Self-worth pertains to self-love or one's positive opinion of himself/herself (Hibbert, 2013), while self-concept is a general image that someone has of him/herself and his/her abilities (Jordan, 2020). Moreover, self-confidence concerns one's trust in himself/herself and his/her ability to cope with setbacks, solve problems, and engage with the environment (Akbari and Sahibzada, 2020). Self-compassion means being kind and forgiving to ourselves and avoiding self-criticism (Neff and Dahm, 2015). The next cognate is self-image, which refers to how you see yourself (McLeod, 2008). Furthermore, self-efficacy has to do with one's confidence in doing a task efficiently (Bandura, 1997). Finally, self-competence is about one viewing himself/herself as qualified, effective, and adept in obtaining goals (Ahmed et al., 2022).

### The conceptualization of social support

As a concept limitedly explored in L2 education, social support refers to various aids that a person receives from inner and outer sources (Minghui et al., 2018). Such kinds of support can be affective, physical, socio-economic, and occupational provided by a network of familial and professional members and peers (Siklos and Kerns, 2006). Concerning teachers' social support, research indicate that it can emerge from internal and external sources in the teaching profession (McNall et al., 2015; Pérez-Fuentes et al., 2019). Internal social support for teachers comes from the workplace and the staff (e.g., colleagues, supervisors, students, principals, etc.), while external social support is provided by sources in teachers' private life, namely, family members and friends (Gavish and Friedman, 2010). It is essential to mention that internally provided social support enhances teachers' commitment, wellbeing, and loyalty, while externally provided social support raises their job satisfaction and performance (McNall et al., 2015; Travers, 2017). Although the concept has been vividly defined, it lacks models, frameworks, and components that reflect the nature and complexity of L2 education, which is left to future researchers.

### Empirical studies

Like other psycho-emotional and social constructs in teaching and learning, the three variables of CA, self-esteem, and social support have been empirically investigated. Concerning CA, the results of several explorations reveal that it correlates with job satisfaction (Fiori et al., 2015), career optimism (Tolentino et al., 2014), job loyalty (Rossier et al., 2012), personality traits (Eryilmaz and Kara, 2017), self-efficacy (McLennan et al., 2017), effectiveness (Zee and Koomen, 2016), sustainable development (Chen et al., 2020), emotional intelligence, and psychological wellbeing (Kulbaş and Kara, 2021). Nevertheless, the impact of EFL/ESL teachers' CA on their self-esteem and the reverse has not been explored to date. Self-esteem itself has rather stronger literature in L2 research in comparison to CA and social support. Accordingly, there have been several studies on the relationship among self-esteem, anxiety, achievement, positive emotions, academic motivation persistence, and flexibility (Leary and MacDonald, 2003; Dewaele, 2011; Basco and Han, 2016; Moriya, 2019). Furthermore, self-esteem has been explored in relation to different language skills and sub-skills in the past decade including speaking (Satriani, 2019), reading (Stranovska and Gadusova, 2020), writing (Fahim and Rad, 2012), and listening (Itzchakov and Weinstein, 2021). Although these are insightful investigations in L2 education, the interplay of self-esteem and psycho-social and occupational variables has not been given due attention.

One such variable is social support, which has entered L2 research and practice in the past couple of years. Research approves that social support is an essential factor for increasing teachers' self-efficacy, psychological wellbeing, teaching effectiveness, job satisfaction, job performance, emotional intelligence, work engagement, commitment, and reducing/removing burnout (Shen, 2009; Field and Buitendach, 2012; Travers, 2017; Minghui et al., 2018; Fiorilli et al., 2019, among others). However, the role of EFL/ESL teachers' social support in generating and enhancing their self-esteem and CA or the effect of CA and self-esteem on the level of social support of the teachers has not been clarified yet. This study argues that, among these three variables, there exists an interplay that each can cause, facilitate, or even be the outcome of the other(s). Trying to theoretically spread the seeds of this domain, the present mini-review is insightful for the body of knowledge on the impact of teacher psychology factors on L2 education.

### Concluding comments and implications

This study presented a theoretical analysis of the possible interaction and interplay of three important psycho-emotional and social variables related to L2 teachers. In the light of the review, it was argued that EFL/ESL teachers' CA, self-esteem, and social support have a strong, three-way relationship, in that they can affect and be affected by one another. A teacher who has a high level of CA and self-esteem would experience more social support from his/her colleagues and staff at the workplace. On the other hand, a teacher with high social support from his/her family and colleagues may feel more self-esteem and CA because all psycho-social factors may change due to interactions and social bonds that a person has. Based on these, it is contended that the article is beneficial for EFL/ESL teachers, teacher trainers, school principals, and researchers. Teachers can use this study to improve their view and awareness of psycho-social factors influential in their profession and of the necessity of CA in their job. Teacher trainers can find this article helpful in offering courses, seminars, and workshops developing EFL/ESL teachers' self-esteem, CA, social support, and ways through which these goals are achieved. Along with pedagogical suggestions, teacher trainers can inform EFL/ESL teachers about the criticality of many inner factors in L2 education. Likewise, school principals can use this study as a guide to creating a friendly workplace for teachers to increase CA, self-esteem, and social support. Finally, L2 researchers may benefit from this mini-review and run future studies to fill the existing gaps in this area and move the field forward. Most of the studies on these three variables are correlational, one-shot, and quantitative. Hence, future studies are recommended to use longitudinal and qualitative designs. The impact of cultural and contextual variations on the claimed interplay of CA, self-esteem, and social support is also missing in the literature. Running empirical studies to statistically approve the interplay of the three constructs is also an interesting topic for research. Moreover, future scholars are recommended to make attempts to provide a disciple-specific model for developing EFL/ESL teachers' CA.

## Author contributions

The author confirms being the sole contributor of this work and has approved it for publication.

## Conflict of interest

The author declares that the research was conducted in the absence of any commercial or financial relationships that could be construed as a potential conflict of interest.

## Publisher's note

All claims expressed in this article are solely those of the authors and do not necessarily represent those of their affiliated organizations, or those of the publisher, the editors and the reviewers. Any product that may be evaluated in this article, or claim that may be made by its manufacturer, is not guaranteed or endorsed by the publisher.
